# Involvement of sodium–glucose cotransporter-1 activities in maintaining oscillatory Cl^−^ currents from mouse submandibular acinar cells

**DOI:** 10.1007/s00360-024-01532-w

**Published:** 2024-02-03

**Authors:** Misa Takeyasu, Katsuyuki Kozai, Makoto Sugita

**Affiliations:** 1 Department of Physiology and Oral Physiology, Graduate School of Biomedical and Health Sciences, Hiroshima University, Kasumi 1-2-3, Minami-ku, Hiroshima, 734-8553 Japan; 2 Department of Pediatric Dentistry, Graduate School of Biomedical and Health Sciences, Hiroshima University, Hiroshima, Japan

**Keywords:** Salivary secretion, Submandibular gland, Gramicidin, Diabetes mellitus

## Abstract

In salivary acinar cells, cholinergic stimulation induces elevations of cytosolic [Ca^2+^]_i_ to activate the apical exit of Cl^−^ through TMEM16A Cl^−^ channels, which acts as a driving force for fluid secretion. To sustain the Cl^−^ secretion, [Cl^−^]_i_ must be maintained to levels that are greater than the electrochemical equilibrium mainly by Na^+^-K^+^-2Cl^−^ cotransporter-mediated Cl^−^ entry in basolateral membrane. Glucose transporters carry glucose into the cytoplasm, enabling the cells to produce ATP to maintain Cl^−^ and fluid secretion. Sodium–glucose cotransporter-1 is a glucose transporter highly expressed in acinar cells. The salivary flow is suppressed by the sodium–glucose cotransporter-1 inhibitor phlorizin. However, it remains elusive how sodium–glucose cotransporter-1 contributes to maintaining salivary fluid secretion. To examine if sodium–glucose cotransporter-1 activity is required for sustaining Cl^−^ secretion to drive fluid secretion, we analyzed the Cl^−^ currents activated by the cholinergic agonist, carbachol, in submandibular acinar cells while comparing the effect of phlorizin on the currents between the whole-cell patch and the gramicidin-perforated patch configurations. Phlorizin suppressed carbachol-induced oscillatory Cl^−^ currents by reducing the Cl^−^ efflux dependent on the Na^+^-K^+^-2Cl^−^ cotransporter-mediated Cl^−^ entry in addition to affecting TMEM16A activity. Our results suggest that the sodium–glucose cotransporter-1 activity is necessary for maintaining the oscillatory Cl^−^ secretion supported by the Na^+^-K^+^-2Cl^−^ cotransporter activity in real time to drive fluid secretion. The concerted effort of sodium–glucose cotransporter-1, Na^+^-K^+^-2Cl^−^ cotransporter, and apically located Cl^−^ channels might underlie the efficient driving of Cl^−^ secretion in different secretory epithelia from a variety of animal species.

## Introduction

In salivary acinar cells, elevations of [Ca^2+^]_i_ evoked by cholinergic agonists stimulate fluid secretion by activating the Ca^2+^-dependent Cl^−^ channel, TMEM16A, in the apical membrane as well as Ca^2+^-dependent K^+^ channels in the basolateral membrane (Lee et al. 2012; Melvin et al. 2005; Proctor 2016). The apical exit of Cl^−^ through the Cl^−^ channels drives fluid secretion (Catalán et al. 2015), while Cl^−^ ions enter the cytoplasm against its electrochemical gradient mainly via a Na^+^-K^+^-2Cl^−^ cotransporter in the basolateral membrane, dependent on the Na^+^ gradient maintained by the activity of Na^+^-K^+^ ATPase (Evans et al. 2000; Sugita et al. 2004). Secretion of negatively charged Cl^−^ ions to the luminal side is followed by the flux of positively charged Na^+^ ions from the interstitium to the lumen via a paracellular pathway. Then water is transported from the interstitium to the lumen via transcellular and paracellular pathways, depending on the osmolarity changes induced by Cl^−^ and Na^+^ secretion to the luminal side (Lee et al. 2012; Melvin et al. 2005; Proctor 2016). Thus, the Cl^−^ secretion via TMEM16A acts as a driving force for fluid secretion. Glucose taken up into the cytoplasm by glucose transporters may be utilized to produce ATP available for maintaining the activities of Na^+^-K^+^ ATPase and Ca^2+^ pumps, and thus for sustaining Cl^−^ and fluid secretion (Zeuthen et al. 2001; Jurysta et al. 2013; Cetik et al. 2014a).

Xerostomia is often observed as the oral symptom of diabetes (Malicka et al. 2014; Lin et al. 2002; Moore et al. 2001). However, the mechanism underlying how diabetes can induce xerostomia remains elusive at a molecular or cellular level. Among glucose transporter genes, sodium–glucose cotransporter-1 is reported to be highly expressed in salivary acinar cells (Jurysta et al. 2012; Cetik et al. 2014b) to transport glucose into the cytoplasm using the Na^+^ electrochemical gradient across the membrane (Hirayama et al. 2007; Díez-Sampedro 2009). It is likely that in the diabetic animal model, the sodium–glucose cotransporter-1 expression in the basolateral membrane of salivary acinar cells is decreased in association with the diminished activities of protein kinase A, and that this resulted from remarkable reduction in sympathetic activity to salivary glands (Sabino-Silva et al. 2010). Nevertheless, it remained unclear whether reduction of the sodium–glucose cotransporter-1 expression and activity in acinar cells is involved in impaired fluid secretion.

Using an ex vivo submandibular gland perfusion technique, we previously characterized fluid secretion induced by the cholinergic agonist carbachol, which depended on extracellular glucose concentration (Terachi et al. 2018). Lowering the extracellular glucose concentration to less than 2.5 mM decreased the carbachol-induced fluid secretion (Terachi et al. 2018). The carbachol-induced salivary flow was suppressed by the sodium–glucose cotransporter-1 inhibitor phlorizin, suggesting that sodium–glucose cotransporter-1 activity or sodium–glucose cotransporter-1-mediated glucose uptake in acinar cells is required to maintain the fluid secretion driven by Cl^−^ secretion in real time (Terachi et al. 2018). However, it remains elusive how sodium–glucose cotransporter-1 contributes to sustaining salivary fluid secretion.

To determine if sodium–glucose cotransporter-1 activity plays a key role in maintaining Cl^−^ secretion to drive fluid secretion, and to clarify how sodium–glucose cotransporter-1 regulates Cl^−^ secretion, we analyzed Cl^−^ currents activated by carbachol in submandibular acinar cells using the gramicidin-perforated patch recording configuration in comparison with those under the conventional whole-cell patch configuration. In the conventional whole-cell patch configuration, the whole-cell Cl^−^ current increased by agonists of interest simply reflects opening of Cl^−^ channels activated by them because Cl^−^ concentration inside the recorded cell is clamped at the same concentration as the patch pipette solution with high Cl^−^ applied for measuring Cl^−^ current. In sharp contrast, the Cl^−^ current recorded in the gramicidin-perforated patch configuration is dependent on Cl^−^ entry through the cohort of Cl^−^ transporters expressed in the recorded cell since gramicidin added to the pipette solution can be incorporated into the patched cell membrane to create monovalent cation-selective pores that are completely impermeable to Cl^−^ (Sugita et al. 2004). Thus, the use of gramicidin in the perforated patch technique enables us to perform whole-cell electrophysiological analyses with normal cellular composition of intracellular Cl^−^ maintained by functionally intact Cl^−^ transporters.

Bumetanide, a loop diuretic, inhibits the ion transport activity of Na^+^-K^+^-2Cl^−^ cotransporter by binding to the amino acid residues of transmembrane helices that make up the faces of a translocation pathway opening to the extracellular surface (Haas and Forbush 2000; Russell 2000; Somasekharan et al. 2012). In our previous study, application of bumetanide remarkably decreased carbachol-activated Cl^−^ currents in rat submandibular acinar cells under the gramicidin-perforated patch configuration (Sugita et al. 2004), suggesting that Na^+^-K^+^-2Cl^−^ cotransporter is the primary Cl^−^ uptake pathway to support Cl^−^ secretion in this cell type. Here we studied the effect of bumetanide on carbachol-activated Cl^−^ currents recorded from mouse submandibular acinar cells under the same configuration to evaluate whether carbachol induces Cl^−^ secretion, substantially dependent upon Cl^−^ entry through Na^+^-K^+^-2Cl^−^ cotransporter in mice. Then we compared the effect of phlorizin on the Cl^−^ currents in the gramicidin-perforated patch recording with that in the whole-cell patch recording to gain insight into how the sodium–glucose cotransporter-1 activity is involved in maintaining Cl^−^ secretion. Furthermore, we discuss how our findings on cooperation of sodium–glucose cotransporter-1 with Na^+^-K^+^-2Cl^−^ cotransporter and apically located Cl^−^ channels in mouse submandibular acinar cells may relate to other cell types and animals.

## Materials and methods

### Materials

Carbachol and collagenase type IV were obtained from Nacalai Tesque and Worthington, respectively. Bovine serum albumin (fraction V), bumetanide, poly-L-lysine, and phlorizin dehydrate were purchased from Sigma-Aldrich.

### Animal experiments’ design

Animal experiments were carried out in accordance with the guidelines laid down by the National Institute of Health in the USA regarding the care and use of animals for experimental procedures. This study was reviewed and approved by the Committee of Research Facilities for Laboratory Animal Science, Hiroshima University, from social and scientific perspectives under “Fundamental Guidelines for Proper Conduct of Animal Experiment and Related Activities in Academic Research Institutions (Ministry of Education, Culture, Sports, Science and Technology, Japan)”. The animals were treated in accordance with “Regulations for Animal Experiments and Related Activities at Hiroshima University”.

Adult male C57BL/6 mice were housed on a 12 h light/dark cycle. Mice had access to water and food ad libitum before the experiments. All mice (2–4 months old) were included and used to isolate submandibular acinar cells for either gramicidin-perforated patch or whole-cell patch recording. In both the gramicidin-perforated patch and whole-cell patch configurations, the data were obtained as current traces recorded from isolated acinar cells. Each current trace was sequentially determined first in the absence of carbachol as control, second in the presence of carbachol to measure the carbachol-induced currents in comparison with control, and third by adding the sodium–glucose cotransporter-1 inhibitor phlorizin or the Na^+^-K^+^-2Cl^−^ cotransporter inhibitor bumetanide with carbachol to examine its effect on the carbachol-induced currents. Then the amplitudes of, and the phlorizin’s effect on carbachol-induced currents were compared between the gramicidin-perforated patch and whole-cell patch configurations. In these patch-clamp analyses, we can start recording the current trace only after obtaining a gigaseal and establishing the gramicidin-perforated or whole-cell recording mode for the recorded cell. Therefore, the analyses obligate us to exclude the data from acinar cells that were not successful in establishing the recording modes. In addition, we needed to exclude the data from acinar cells with the gigaseal when the seal was broken during the application of carbachol and phlorizin or bumetanide due to inability of comparison among their experimental conditions.

### Gramicidin-perforated patch recording from isolated acinar cells

Gramicidin-perforated patch recording was performed as described previously (Sugita et al. 2000, 2004). Submandibular glands were removed from male C57BL/6 mice euthanized with intraperitoneal injection of sodium pentobarbital. The minced glands were digested for 12 min at 37 °C with collagenase type IV (2 mg/ml) dissolved in modified Hanks’ balanced salt solution (HBSS) (137 mM NaCl, 5.4 mM KCl, 2 mM CaCl_2_, 0.49 mM MgCl_2_, 0.41 mM MgSO_4_, 0.34 mM Na_2_HPO_4_, 0.44 mM KH_2_PO_4_, 10 mM glucose, and 0.1% bovine serum albumin (pH 7.4)). The digestives were dispersed by pipetting, and then filtered through a 150 μm nylon mesh to remove tissue clumps. The cells were then filtered with a 20 μm nylon mesh. The 20–150 μm fraction was centrifuged, and the pellet resuspended in modified HBSS was used as an isolated acinar cell fraction. The isolated acinar cells were allowed to stick onto coverslips coated with poly-L-lysine. The acinar cells on the cover glass were placed in the chamber, which was constantly perfused with the modified HBSS without 0.1% bovine serum albumin.

Gramicidin-perforated patch recording was performed at room temperature (25 °C). Patch-clamp electrodes were pulled from borosilicate glass capillaries. The gramicidin-perforated patch pipette solution contained 150 mM KCl and 10 mM HEPES (adjusted to pH 7.4 with KOH). Gramicidin was first dissolved in methanol to a concentration of 10 mg/ml, and then diluted in the pipette solution to a final concentration of 200 μg/ml immediately before use. Before backfilling the pipette with the gramicidin-containing solution, the pipette tip was filled with gramicidin-free pipette solution by a brief immersion. After obtaining a gigaseal, the command potential was set at − 40 mV, so that after perforation, the cell was roughly at resting potential. Gramicidin-perforated patch recording was started after the stabilization of the capacitive currents. Ionic currents were measured under voltage clamping at the holding potential of − 80 mV, a value close to the potassium equilibrium potential. The ionic currents were measured with an Axopatch 200B amplifier (Axon instruments), low-pass filtered at 5 kHz, sampled at 10 kHz, digitized with a Digidata 1200B interface (Axon instruments), and recorded directly onto a hard disk using pClamp 9 software (Axon instruments).

The average values of the current amplitude were determined using LabChart 7 (AD Instruments). Oscillatory events of Cl^−^ currents were detected and analyzed using LabChart 7. We measured the base lines of current traces, and the integral and the peak amplitudes of oscillatory Cl^−^ currents which were calculated from the baseline currents using computer-assisted manual detection on the LabChart 7 software. The peak amplitude was calculated from the maximum of every inward current spike which was measured during the indicated period (30 s) and averaged.

Results are presented as means with SD of *n* observations. Paired or unpaired Student’s *t* test was used for pairwise sample comparison to determine statistical significance. Statistical analyses for comparison of data among three conditions in a sequence of applications of carbachol and phlorizin (or bumetanide) were performed by one-way ANOVA, followed by post hoc Ryan’s method that was carried out for comparisons between all pairs. *P* values < 0.05 were considered statistically significant.

### Whole-cell patch recording from isolated acinar cells

Conventional whole-cell patch recording was carried out as described previously (Hirono et al. 1998; Sugita et al. 2004). The pipette solution contained 140 mM KCl, 1 mM MgCl_2_, 10 mM HEPES, 0.5 mM ethylene glycol-bis(ß-aminoethyl ether)-*N,N,N',N'*-tetraacetic acid (EGTA), 10 mM glucose, and 1 mM ATP (adjusted to pH 7.4 with KOH). The isolated acinar cells on the cover glass were placed in the chamber, which was constantly perfused with the modified HBSS without 0.1% bovine serum albumin. To establish whole-cell recording, additional suction was performed to rupture the patch membrane after the gigaseal formation. Ionic currents were measured under voltage clamping at the holding potential of − 80 mV, a value close to the potassium equilibrium potential. The ionic currents were measured with an Axopatch 200B amplifier (Axon instruments), low-pass filtered at 5 kHz, sampled at 10 kHz, digitized with a Digidata 1200B interface (Axon instruments), and recorded directly onto a hard disk using pClamp 9 software (Axon instruments). Then we determined the average values of the current amplitude, the base lines of current traces (non-oscillatory inward currents), and the integral, and the peak amplitudes of oscillatory Cl^−^ currents as described above in the section of gramicidin-perforated patch recording.

## Results

### Comparison of carbachol-induced Cl^−^ currents between the whole-cell patch and the gramicidin-perforated patch configurations

**Fig. 1:**
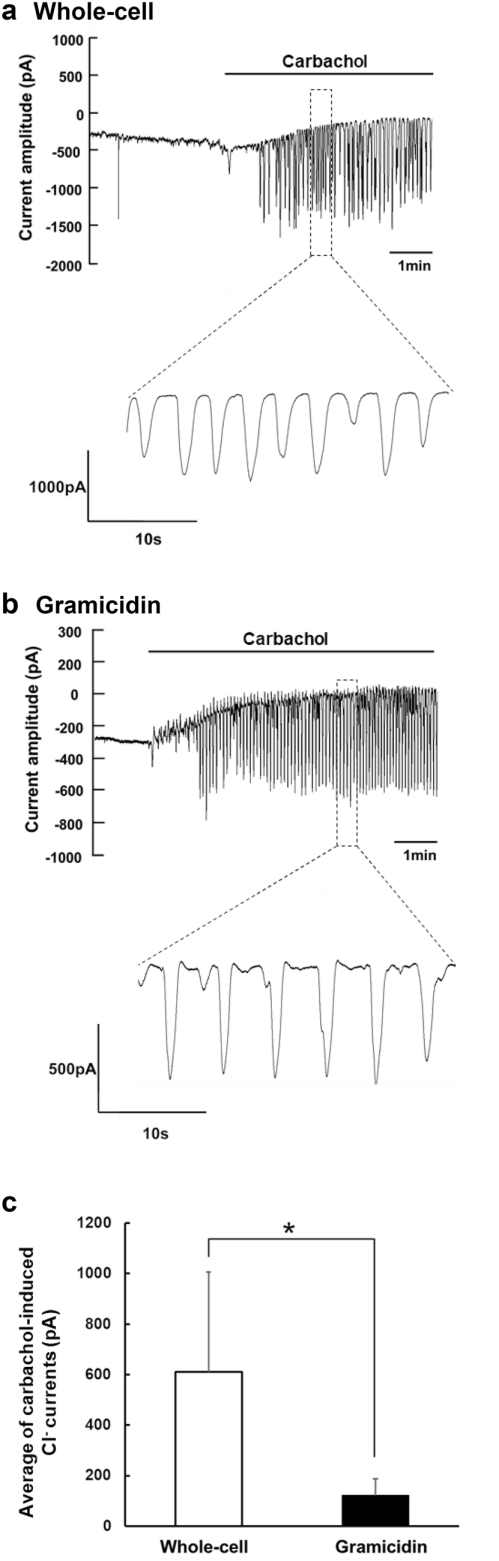
Carbachol-induced Cl^−^ currents recorded in whole-cell and gramicidin-perforated patch configurations. **a** Carbachol-induced Cl^−^ currents in the conventional whole-cell patch configuration were measured in submandibular acinar cells that were voltage clamped at − 80 mV. Carbachol (300 nM) was added to the perfusate during the period indicated by the horizontal bar. The time-resolved trace of carbachol-induced repetitive spikes of inward current is also shown. **b** Carbachol(300 nM)-induced Cl^−^ currents were recorded in the gramicidin-perforated patch configuration in acinar cells voltage clamped at − 80 mV. **c** Comparison of carbachol-induced Cl^−^ currents between the whole-cell patch (whole cell) and gramicidin-perforated patch (gramicidin) configurations. The average currents were determined in the 30 s showing steady baseline currents within 3.5—5 min after adding carbachol since oscillatory Cl^−^ currents induced by carbachol gradually increased during the initial 3 min after the addition of carbachol, and became constant thereafter. Although the carbachol-induced Cl^−^ current represents a negative value because of that measured as inward current, we converted the negative value to the positive one to have their comparison simpler and more convenient. Results are presented as means with error bars representing SD (*n* = 5 for Whole-cell, and 9 for Gramicidin). *P < 0.05 in the unpaired Student’s *t* test

First, submandibular acinar cells were voltage clamped at − 80 mV, a value close to the K^+^ equilibrium potential, in the conventional whole-cell patch configuration. Carbachol induced repetitive spikes of inward current expected to be Ca^2+^-activated Cl^−^ current via TMEM16A (Fig. 1a). In the gramicidin-perforated patch configuration, the ionic currents were also measured at the holding potential of −80 mV. Figure 1b shows a representative trace of the ionic current during carbachol application that similarly elicited the oscillatory inward current expected to be Ca^2+^-activated Cl^−^ current. Of note, the average value of carbachol-induced Cl^−^ currents recorded in the gramicidin-perforated patch configuration was much lower than that observed in the whole-cell patch configuration (Fig. 1c).

**Fig. 2:**
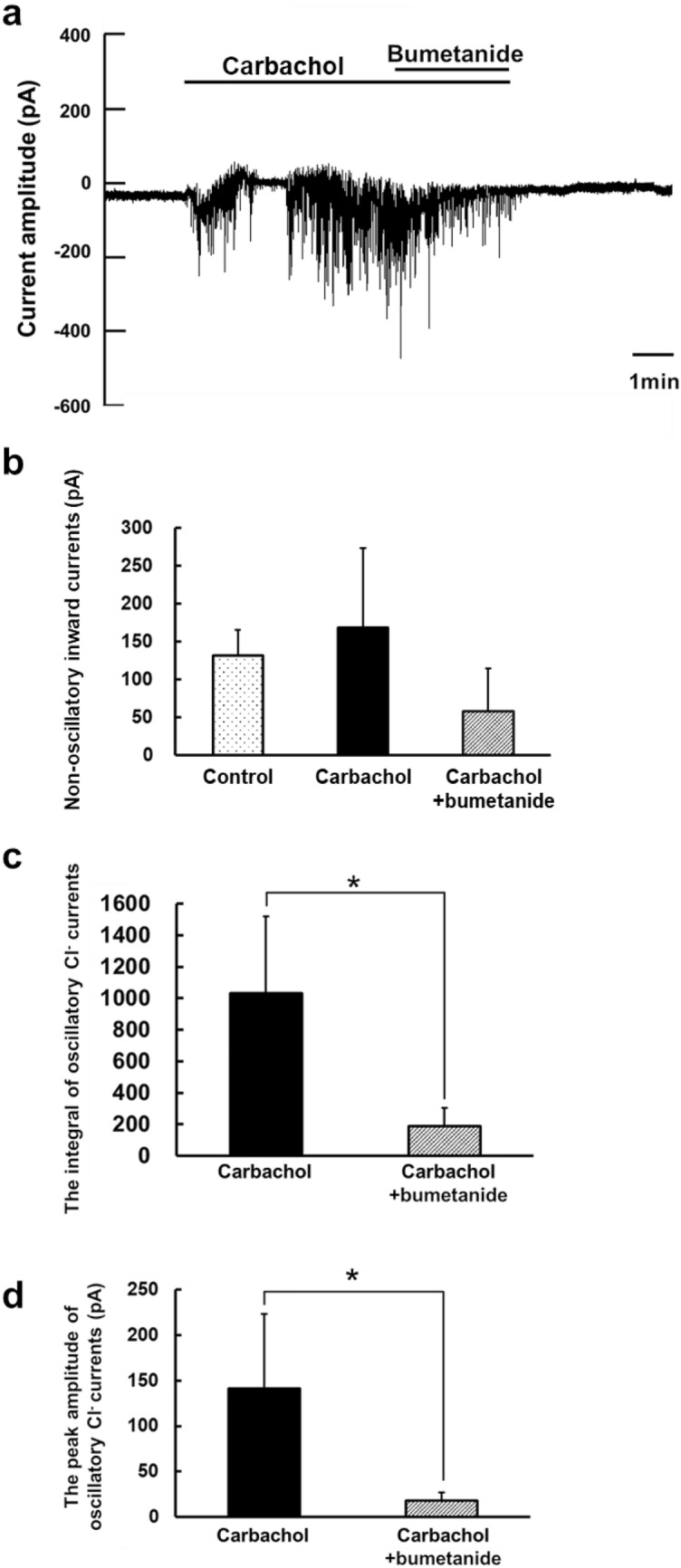
The effect of bumetanide on carbachol-induced Cl^−^ currents in the gramicidin-perforated patch configuration. **a** A representative trace of the Cl^−^ current from isolated acinar cells in the gramicidin-perforated patch configuration during the application of carbachol (300 nM) and bumetanide (500 μM) as indicated by the horizontal bars. **b** The effect of bumetanide on non-oscillatory inward currents (baseline current). **c** The effect of bumetanide on the integral of oscillatory Cl^−^ currents. **d** The effect of bumetanide on the peak amplitude of oscillatory Cl^−^ currents. The averages of non-oscillatory inward currents **b**, the integral **c**, and the peak amplitude **d** were determined in the 30 s showing steady baseline currents within 0–3 min before switching the perfusate. Although the non-oscillatory inward current, the integral and the peak amplitude of oscillatory Cl^−^ current represent negative values because of those measured as inward current, we converted the negative values to the positive values to have their comparison simpler. The values obtained in the presence of carbachol (carbachol), and in the sequential addition of bumetanide (carbachol + bumetanide) were compared. Results are presented as means with error bars representing SD (*n* = 5). *P < 0.05 in the paired Student’s *t* test for **c** and **d**

The carbachol-induced Cl^−^ current recorded in the whole-cell patch configuration represents the Cl^−^ exit via opened Cl^−^ channels under the constant cytosolic concentration of Cl^−^ supplied from the patch-clamp pipette, and simply reflects the changes in activity of TMEM16A responding to the application of carbachol. However, since gramicidin creates monovalent cation-selective pores, the carbachol-induced current in the gramicidin-perforated patch recording represents the Cl^−^ exit via Cl^−^ channels dependent on Cl^−^ entry through Cl^−^ transporters expressed in the cells. Therefore, to sustain the Cl^−^ current in the gramicidin-perforated patch configuration, [Cl^−^]_i_ must be maintained in the cytoplasm above its electrochemical equilibrium value by Cl^−^ transporters. Addition of bumetanide, an inhibitor of Na^+^-K^+^-2Cl^−^ cotransporter, markedly reduced the carbachol-induced Cl^−^ current when analyzing the integral and peak amplitude of oscillatory Cl^−^ currents induced by carbachol (Fig. 2). Thus, these data suggested that the carbachol-induced Cl^−^ current from mouse submandibular acinar cells under the gramicidin-perforated patch configuration represented the Cl^−^ efflux via TMEM16A dependent on Cl^−^ entry mainly through Na^+^-K^+^-2Cl^−^ cotransporter.

### The effect of sodium–glucose cotransporter inhibition on carbachol-induced Cl^−^ currents

Our previous data indicated that the sodium–glucose cotransporter-1 inhibitor phlorizin partially inhibited the carbachol-induced fluid secretion without reducing the carbachol-induced increase in [Ca^2+^]_i_ (Terachi et al. 2018). It suggests that phlorizin may inhibit the carbachol-induced fluid secretion by altering the activities of ion channels and transporters downstream of [Ca^2+^]_i_ signals. To determine if sodium–glucose cotransporter-1 activity or sodium–glucose cotransporter-1-mediated glucose entry is required for maintaining Cl^−^ secretion to drive fluid secretion, we analyzed the effect of phlorizin on carbachol-activated Cl^−^ currents in submandibular acinar cells using the whole-cell patch and the gramicidin-perforated patch configurations.

**Fig. 3:**
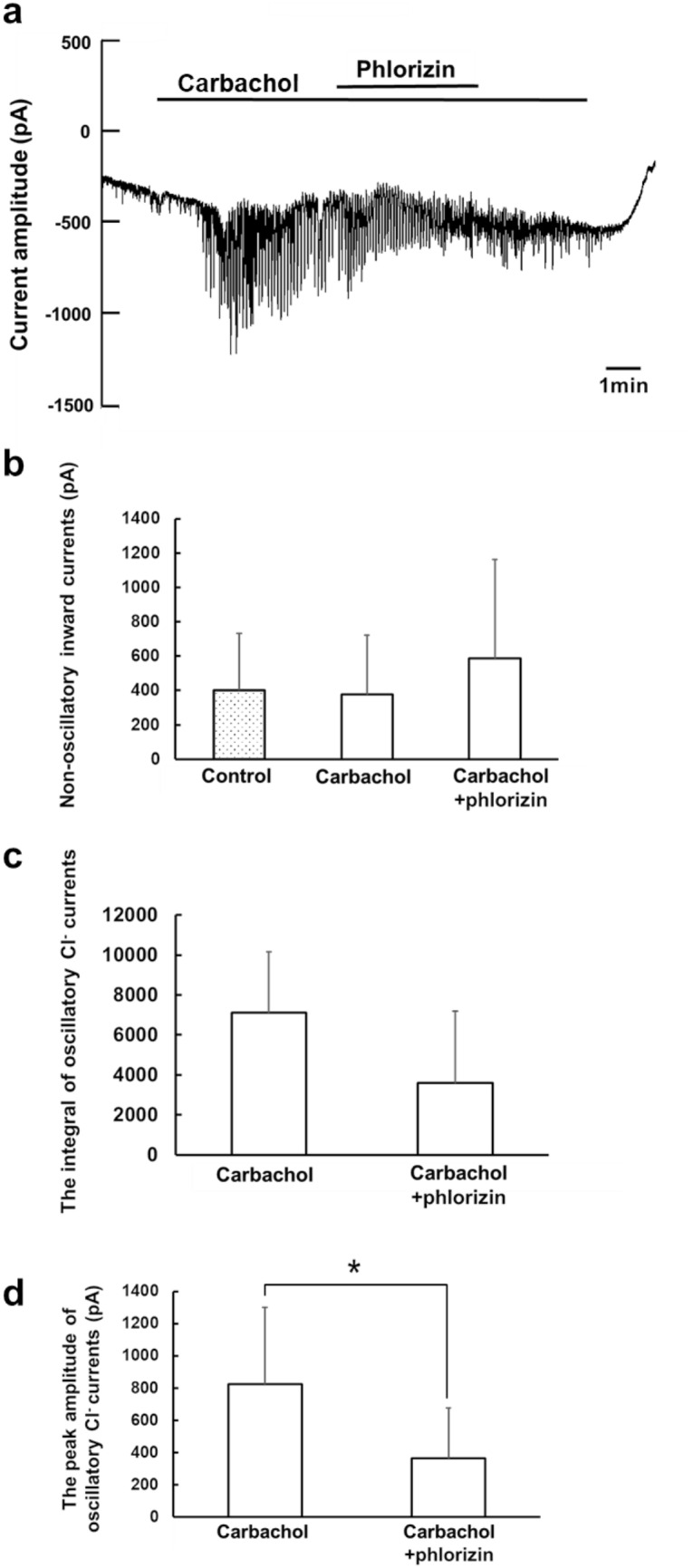
The effect of phlorizin on carbachol-induced Cl^−^ currents in the whole-cell patch configuration. **a** A representative trace of the Cl^−^ current from isolated acinar cells in the whole-cell patch configuration during the application of carbachol (300 nM) and phlorizin (100 μM) as indicated by the horizontal bars. **b** The effect of phlorizin on non-oscillatory inward currents (baseline current). **c** The effect of phlorizin on the integral of oscillatory Cl^−^ currents. **d** The effect of phlorizin on the peak amplitude of oscillatory Cl^−^ currents. The averages of non-oscillatory inward currents **b**, the integral **c**, and the peak amplitude **d** were determined as described earlier in Fig. 2. The values obtained in the presence of carbachol (carbachol), and in the sequential addition of phlorizin (carbachol + phlorizin) were compared. Results are presented as means with error bars representing SD (*n* = 5). *P < 0.05 in the paired Student’s *t* test for **d**

First, we evaluated the effect of phlorizin on carbachol-activated Cl^−^ currents in the conventional whole-cell patch configuration, where changes in whole-cell Cl^−^ current simply reflect alteration of the Cl^−^ channel activities (Fig. 3). Addition of phlorizin following carbachol application had little effect on non-oscillatory inward currents shown as changes in baseline currents, representing no statistical difference between carbachol and carbachol + phlorizin (Fig. 3b). When deducing and comparing the integral of oscillatory Cl^−^ current (inward current) calculated from the baseline current, the addition of phlorizin following carbachol did not lead to a significant change in the integral of oscillatory Cl^−^ currents induced by carbachol, showing no statistical difference between carbachol and carbachol + phlorizin (Fig. 3c). However, we found that the peak amplitudes of oscillatory Cl^−^ currents calculated from the baseline currents were significantly different between carbachol and carbachol + phlorizin (Fig. 3d). Addition of phlorizin reduced the peak amplitude of oscillatory Cl^−^ currents induced by carbachol. Therefore, these results suggest that the sodium–glucose cotransporter-1 inhibition may partially suppress the channel activity of TMEM16A, which is primarily regulated by elevation of [Ca^2+^]_i_, without reducing the carbachol-induced increase in [Ca^2+^]_i_ (Terachi et al. 2018).

**Fig. 4:**
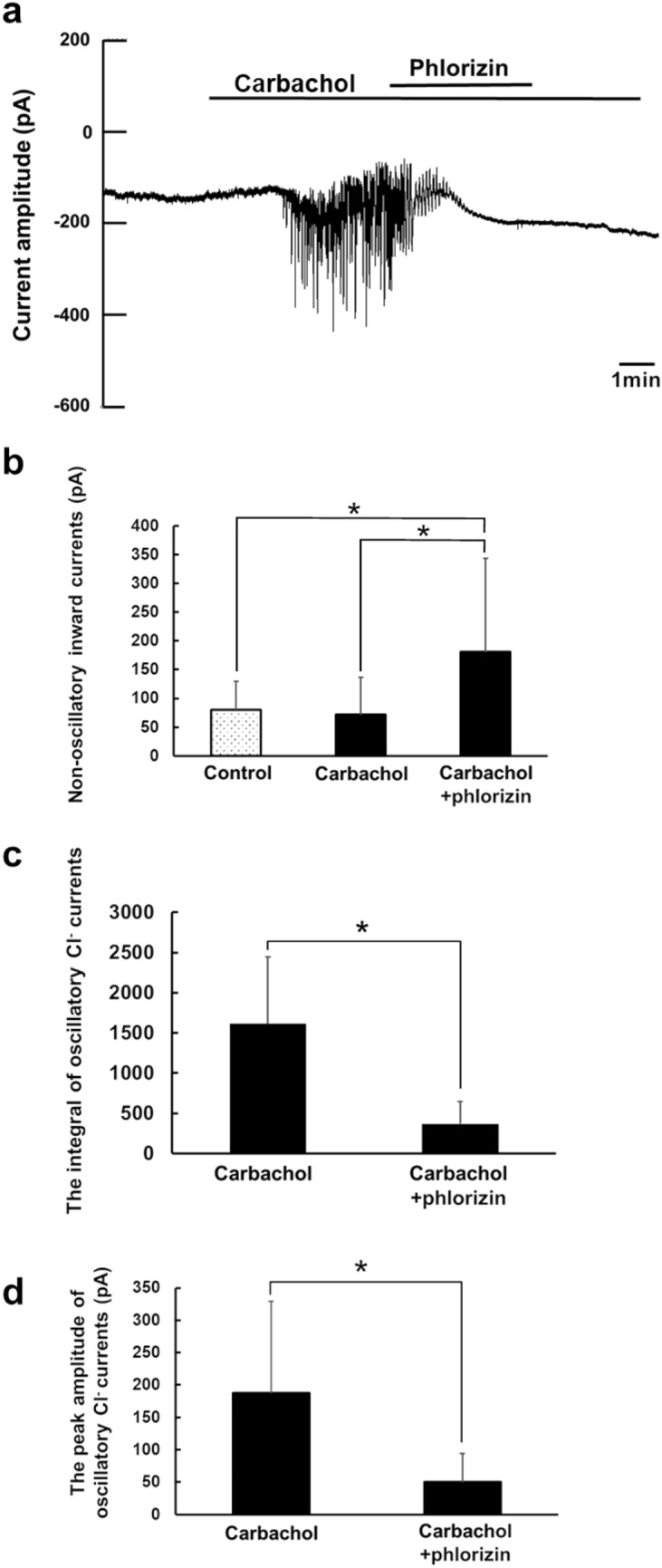
The effect of phlorizin on carbachol-induced Cl^−^ currents in the gramicidin-perforated patch configuration. **a** A representative trace of the Cl^−^ current from isolated acinar cells in the gramicidin-perforated patch configuration during the application of carbachol (300 nM) and phlorizin (100 μM). **b** The effect of phlorizin on non-oscillatory inward currents (baseline current). **c** The effect of phlorizin on the integral of oscillatory Cl^−^ currents. **d** The effect of phlorizin on the peak amplitude of oscillatory Cl^−^ currents. The averages of non-oscillatory inward currents **b**, the integral **c**, and the peak amplitude **d** were determined and compared as described earlier in Fig. 3. Results are presented as means with error bars representing SD (*n* = 9). *P < 0.05 in one-way ANOVA followed by post hoc Ryan’s method for **b**, and in the paired Student’s *t* test for **c** and **d**

Second, we examined the effect of phlorizin on carbachol-activated Cl^−^ currents in the gramicidin-perforated patch configuration, where the Cl^−^ current is carried dependent on Cl^−^ entry through membrane transporters (Fig. 4). Addition of phlorizin following carbachol increased non-oscillatory inward current shown as changes in baseline currents (Fig. 4b). Importantly, the phlorizin addition significantly reduced both the integral and peak amplitude of oscillatory Cl^−^ currents induced by carbachol (Fig. 4c, d), suggesting that the sodium–glucose cotransporter-1 inhibition may reduce the Cl^−^ efflux via TMEM16A dependent on Cl^−^ entry through the Na^+^-K^+^-2Cl^−^ cotransporter.

When the relative integral was calculated as a ratio of the integral of oscillatory Cl^−^ currents in the presence of phlorizin (carbachol + phlorizin) compared with that in the absence of phlorizin (carbachol) as 1 for each experiment, the averages of the relative integral values for the whole-cell patch and the gramicidin-perforated patch configurations were 0.48 ± 0.37 (*n* = 5) and 0.23 ± 0.13 (*n* = 9), respectively. There was no statistical difference in the relative integral values of carbachol + phlorizin between the two configurations. When the relative peak amplitude was calculated as a ratio of the peak amplitude of oscillatory Cl^−^ currents in the presence of phlorizin (carbachol + phlorizin) compared with that in the absence of phlorizin (carbachol) as 1 for each experiment, the averages of the relative peak amplitudes for the whole-cell patch and the gramicidin-perforated patch configurations were 0.43 ± 0.23 (*n* = 5) and 0.26 ± 0.12 (*n* = 9), respectively. No statistical difference in the relative peak amplitude values of carbachol + phlorizin was observed between the two configurations.

## Discussion

**Fig. 5:**
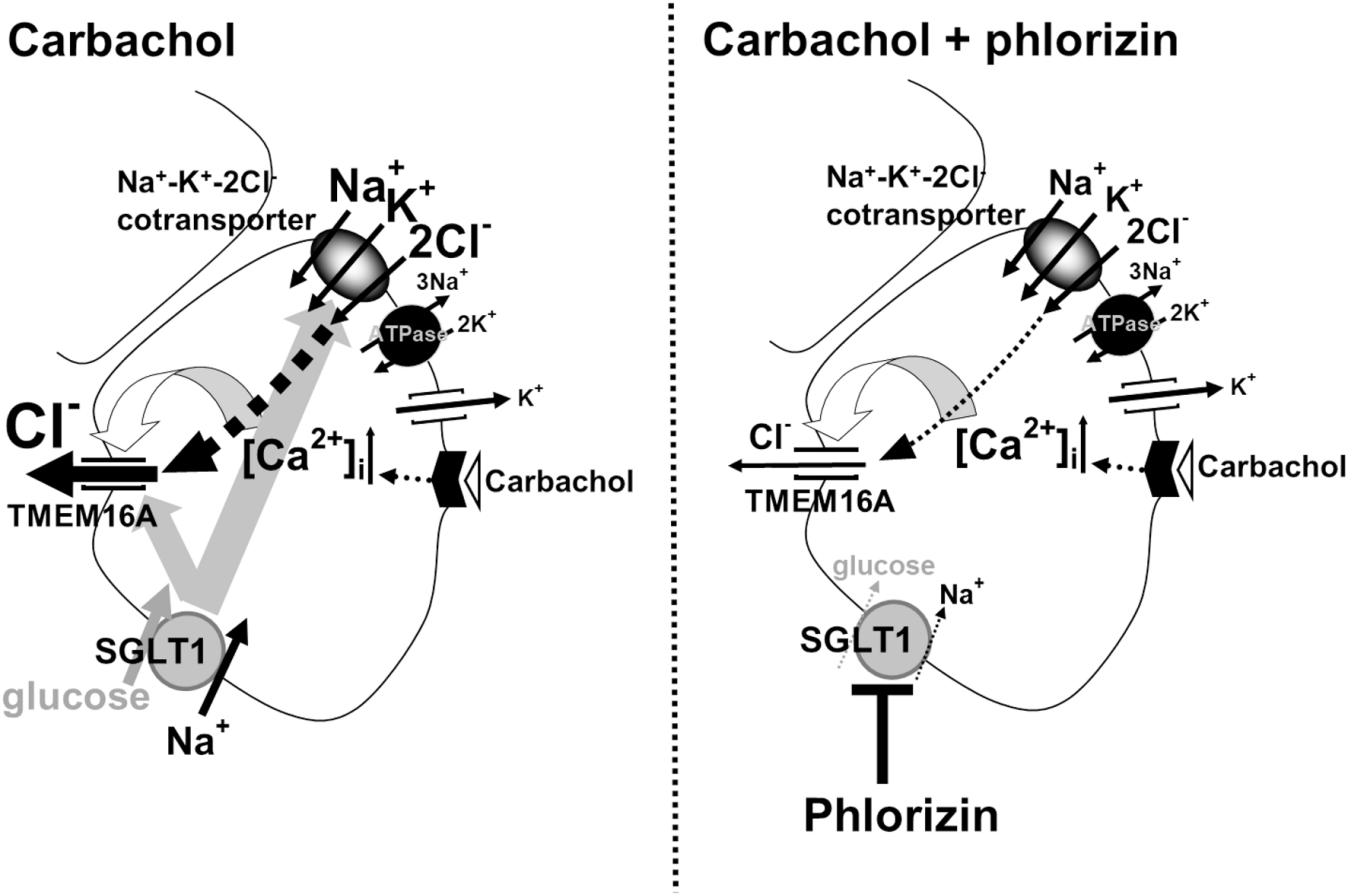
Schematic diagrams of the proposed ionic pathways leading to carbachol-induced Cl^−^ currents in submandibular acinar cells in the presence and the absence of phlorizin. Carbachol-induced Cl^−^ currents in the gramicidin-perforated patch recording are carried by Cl^−^ efflux via the Ca^2+^-activated Cl^−^ channel TMEM16A, dependent upon Cl^−^ entry mainly through Na^+^-K^+^-2Cl^−^ cotransporter. The oscillatory Cl^−^ currents are maintained by the activity of sodium–glucose cotransporter-1 (SGLT1) as the currents are decreased by phlorizin application

Carbachol induces oscillatory Cl^−^ currents for Cl^−^ secretion to drive fluid secretion in rat submandibular acinar cells (Sugita et al. 2004). In the present study, the oscillatory Cl^−^ currents were recorded from mouse submandibular acinar cells in both the whole-cell patch and gramicidin-perforated patch configurations. Of note, the carbachol-induced Cl^−^ current in the whole-cell patch configuration was much greater than that in the gramicidin-perforated patch configuration. This is because the Cl^−^ current recorded in the gramicidin-perforated patch configuration is carried by Cl^−^ efflux via Cl^−^ channels, dependent upon Cl^−^ entry through Cl^−^ transporters expressed in the examined cell, whereas the Cl^−^ current in the whole-cell patch configuration solely reflects the Cl^−^ channel activity. More precisely, in contrast with that oscillatory Cl^−^ currents observed in the whole-cell patch configuration enable us to simply characterize the cellular events of opening and closing of TMEM16A, a Ca^2+^-activated Cl^−^ channel, those recorded in the gramicidin-perforated patch configuration reflects the TMEM16A-mediated efflux of intracellular Cl^−^ which is raised above the electrochemical equilibrium level by membrane transporters. By exhibiting the oscillatory Cl^−^ current sensitive to bumetanide, a Na^+^-K^+^-2Cl^−^ cotransporter inhibitor, in the gramicidin-perforated patch recording, our results suggested that the basolaterally located Na^+^-K^+^-2Cl^−^ cotransporter is the dominant Cl^−^ influx pathway for supporting oscillatory Cl^−^ efflux through the apical Ca^2+^-activated Cl^−^ channel TMEM16A in this configuration (Fig. 5), and also in intact acinar cells as previously observed in rat submandibular acinar cells (Sugita et al. 2004).

Then we examined if sodium–glucose cotransporter-1 activity is required for maintaining oscillatory Cl^−^ currents, and how it is involved in regulation of the Cl^−^ secretion by analyzing the effect of the sodium–glucose cotransporter-1 inhibitor, phlorizin, on carbachol-induced Cl^−^ currents in the whole-cell patch and gramicidin-perforated patch configurations. Phlorizin partially inhibits carbachol-induced fluid secretion without reducing the elevation of [Ca^2+^]_i_ by carbachol, suggesting that it may alter the activities of ion channels and transporters downstream of [Ca^2+^]_i_ signals (Terachi et al. 2018). When comparing the effect of phlorizin on carbachol-induced Cl^−^ currents between the whole-cell patch and gramicidin-perforated patch configurations, first, we could expect the Cl^−^ currents in the gramicidin-perforated patch, but not in the whole-cell patch configuration, to be suppressed by phlorizin if the application of phlorizin inhibits the Na^+^-K^+^-2Cl^−^ cotransporter-mediated Cl^−^ entry without affecting the TMEM16A channel activity. Second, we could expect the Cl^−^ currents in the whole-cell patch, but not in the gramicidin-perforated patch configuration, to be partly suppressed by phlorizin if it can partially inhibit the TMEM16A channel activity without affecting the Na^+^-K^+^-2Cl^−^ cotransporter-mediated Cl^−^ entry because of Na^+^-K^+^-2Cl^−^ cotransporter acting as the rate-limiting step for the Cl^−^ currents. This pattern of results was exemplified by our previous study that showed partial block of the Cl^−^ channel activity had little effect on the carbachol-induced Cl^−^ current recorded under the gramicidin-perforated patch configuration (Sugita et al. 2004). Third, we expect the Cl^−^ currents in both the whole-cell patch and gramicidin-perforated patch configurations to be suppressed by phlorizin if it potently inhibits the TMEM16A channel activity.

Figures 3cd and 4cd show that the oscillatory Cl^−^ currents in the gramicidin-perforated patch configuration (Fig. 4cd) were slightly more sensitive to phlorizin application than those in the whole-cell patch configuration (Fig. 3cd). Therefore, it is likely that the sodium–glucose cotransporter-1 inhibition may suppress the oscillatory dynamics of carbachol-induced Cl^−^ secretion by reducing the Na^+^-K^+^-2Cl^−^ cotransporter-mediated Cl^−^ entry or the Cl^−^ efflux dependent on Cl^−^ entry through Na^+^-K^+^-2Cl^−^ cotransporter, and in addition, by affecting the TMEM16A activity. Thus, our data suggest that the sodium–glucose cotransporter-1 activity or the sodium–glucose cotransporter-1-mediated glucose uptake may be necessary for maintaining the Cl^−^ secretion supported by the Na^+^-K^+^-2Cl^−^ cotransporter activity in real time to drive fluid secretion (Fig. 5).

Our previous data indicated that elimination of extracellular glucose partly decreases the carbachol-induced Cl^−^ currents via Ca^2+^-activated Cl^−^ channels, dependent upon Cl^−^ entry through the Na^+^-K^+^-2Cl^−^ cotransporter, to reduce the carbachol-induced fluid secretion (Terachi et al. 2018). Hence, the glucose uptake mediated by sodium–glucose cotransporter-1 is in part responsible for maintaining the Cl^−^ and fluid secretion. However, it is likely that phlorizin had much more suppressive effect on the carbachol-induced Cl^−^ currents than glucose elimination did. Sodium–glucose cotransporter-1 is reported to carry water and glucose molecules using the energy in the Na^+^ gradient across the membrane with a stoichiometry of two Na^+^, one glucose, and 200–250 water molecules per transport cycle (Loo et al. 1996; Duquette et al. 2001). Therefore, the water transport mediated by sodium–glucose cotransporter-1 may have a modulatory effect on maintaining the carbachol-induced Cl^−^ secretion in association with the glucose transport that can also regulate the secretion in a different manner. With regard to a pathological condition, reduction of sodium–glucose cotransporter-1 expression at the protein level was observed in salivary acinar cells in diabetic animal models (Sabino-Silva et al. 2010). Alterations in expression and function of sodium–glucose cotransporter-1 may partly underlie how diabetic patients suffer from xerostomia as observed in the perfused submandibular glands where fluid secretion was suppressed by phlorizin.

Importantly, since sodium–glucose cotransporter-1, Na^**+**^-K^**+**^-2Cl^−^ cotransporter, and apically located Cl^−^ channels are commonly expressed in diverse secretory epithelia from a variety of animal species, several different secretory epithelia may share the similar regulatory role of sodium–glucose cotransporter-1 in Cl^−^ secretion. Among the three major salivary glands in mice, the protein expression levels of the Ca^2+^-activated Cl^−^ channel TMEM16A were reported to be significantly greater in parotid glands, compared with submandibular and sublingual glands (Kondo et al. 2015). It was also reported that the Na^**+**^-K^**+**^-2Cl^−^ cotransporter activity in submandibular glands was about twice that of parotid glands and more than 12-fold greater than that of sublingual glands (Kondo et al. 2015). The relative contribution of TMEM16A and Na^**+**^-K^**+**^-2Cl^−^ cotransporter to determining the rate of Cl^−^ secretion might be varied among the three major salivary glands. However, since sodium–glucose cotransporter-1 is commonly expressed in the salivary glands (Madunić et al. 2017), the sodium–glucose cotransporter-1 activity or the sodium–glucose cotransporter-1-mediated glucose uptake may also regulate the rate of Cl^−^ efflux via TMEM16A dependent upon Cl^−^ entry through Na^+^-K^+^-2Cl^−^ cotransporter in mouse parotid and sublingual glands. Furthermore, it is likely that the ion transport mechanisms in rat, rabbit, sheep, and human salivary glands are equivalent to those in the mouse (Lee et al. 2005; Lytle et al. 1995; Tarpey et al. 1995; Nakamoto et al. 2007). Therefore, the concerted effort of sodium–glucose cotransporter-1, Na^**+**^-K^**+**^-2Cl^−^ cotransporter and TMEM16A to drive Cl^−^ secretion may be observed in salivary glands of those animal species.

Because a similar coordinated transport process occurs in a variety of secretory epithelia including those of murine and human intestines (Gawenis et al. 2004; Reynolds et al. 2007), and of murine, porcine, and human airways (Gillie et al. 2001; Flores-Delgado et al. 2016; Baines et al. 2023), those secretory epithelia might share the common sodium–glucose cotransporter-1-mediated regulation of Cl^−^ secretion via apically located Cl^−^ channels, dependent on Cl^−^ entry through Na^**+**^-K^**+**^-2Cl^−^ cotransporter. Importantly, shark rectal glands as well as snake salt glands possess similar systems for Cl^−^ secretion, in which basolateral Na^+^-K^+^ ATPase creates a sodium gradient that allows Cl^−^ to enter the gland epithelial cells via basolateral Na^**+**^-K^**+**^-2Cl^−^ cotransporter, and then to be secreted via apically located Cl^−^ channels such as a cystic fibrosis transmembrane conductance regulator (CFTR) (Silva et al. 1977; Hannafin et al. 1983; Babonis and Evans 2011). Of note, Cl^−^ secretion by shark rectal glands is reported to be partly dependent on glucose that is transported into the cells via sodium–glucose cotransporter-1 (Deck et al. 2017; Kinne et al. 2020). Accordingly, the sodium–glucose cotransporter-1-mediated glucose uptake might also help determine the rate of Cl^−^ secretion in shark rectal glands by controlling the Cl^−^ efflux dependent on Cl^−^ entry through Na^+^-K^+^-2Cl^−^ cotransporter.

Collectively, it is now of critical relevance to further clarify how sodium–glucose cotransporter-1 regulates the Cl^−^ secretion to understand a key mechanism underlying generation of the Cl^−^ driving force for fluid secretion in a wide variety of secretory epithelia, and to develop therapeutic means by manipulating the sodium–glucose cotransporter-1 activity for patients suffering from xerostomia and other diseases related to dysfunction in secretory epithelia.

## Data Availability

Data are available on request from the corresponding author.

## References

[CR1] Babonis LS, Evans DH (2011). Morphological and biochemical evidence for the evolution of salt glands in snakes. Comp Biochem Physiol A Mol Integr Physiol.

[CR2] Baines DL, Vasiljevs S, Kalsi KK (2023). Getting sweeter: new evidence for glucose transporters in specific cell types of the airway?. Am J Physiol Cell Physiol.

[CR3] Catalán MA, Kondo Y, Peña-Munzenmayer G, Jaramillo Y, Liu F, Choi S (2015). A fluid secretion pathway unmasked by acinar-specific Tmem16A gene ablation in the adult mouse salivary gland. Proc Natl Acad Sci USA.

[CR4] Cetik S, Rzajeva A, Hupkens E, Malaisse WJ, Sener A (2014). Uptake and metabolism of D-glucose in isolated acinar and ductal cells from rat submandibular glands. Cell Biochem Funct.

[CR5] Cetik S, Hupkens E, Malaisse WJ, Sener A, Popescu IR (2014). Expression and localization of glucose transporters in rodent submandibular salivary glands. Cell Physiol Biochem.

[CR6] Deck CA, Anderson WG, Conlon JM, Walsh PJ (2017). The activity of the rectal gland of the North Pacific spiny dogfish Squalus suckleyi is glucose dependent and stimulated by glucagon-like peptide-1. J Comp Physiol B.

[CR7] Díez-Sampedro A (2009). Involvement of amino acid 36 in TM1 in voltage sensitivity in mouse Na^+^/glucose cotransporter SGLT1. J Membr Biol.

[CR8] Duquette PP, Bissonnette P, Lapointe JY (2001). Local osmotic gradients drive the water flux associated with Na^+^/glucose cotransport. Proc Natl Acad Sci USA.

[CR9] Evans RL, Park K, Turner RJ, Watson GE, Nguyen HV, Dennett MR (2000). Severe impairment of salivation in Na^+^/K^+^/2Cl^-^ cotransporter (NKCC1)-deficient mice. J Biol Chem.

[CR10] Flores-Delgado G, Lytle C, Quinton PM (2016). Site of fluid secretion in small airways. Am J Respir Cell Mol Biol.

[CR11] Gawenis LR, Hut H, Bot AG, Shull GE, de Jonge HR, Stien X, Miller ML, Clarke LL (2004). Electroneutral sodium absorption and electrogenic anion secretion across murine small intestine are regulated in parallel. Am J Physiol Gastrointest Liver Physiol.

[CR12] Gillie DJ, Pace AJ, Coakley RJ, Koller BH, Barker PM (2001). Liquid and ion transport by fetal airway and lung epithelia of mice deficient in sodium-potassium-2-chloride transporter. Am J Respir Cell Mol Biol.

[CR13] Haas M, Forbush B (2000). The Na-K-Cl cotransporter of secretory epithelia. Annu Rev Physiol.

[CR14] Hannafin J, Kinne-Saffran E, Friedman D, Kinne R (1983). Presence of a sodium-potassium chloride cotransport system in the rectal gland of Squalus acanthias. J Membr Biol.

[CR15] Hirayama BA, Loo DD, Díez-Sampedro A, Leung DW, Meinild AK, Lai-Bing M (2007). Sodium-dependent reorganization of the sugar-binding site of SGLT1. Biochemistry.

[CR16] Hirono C, Sugita M, Furuya K, Yamagishi S, Shiba Y (1998). Potentiation by isoproterenol on carbachol-induced K^+^ and Cl^-^ currents and fluid secretion in rat parotid. J Membr Biol.

[CR17] Jurysta C, Nicaise C, Cetik S, Louchami K, Malaisse WJ, Sener A (2012). Glucose transport by acinar cells in rat parotid glands. Cell Physiol Biochem.

[CR18] Jurysta C, Nicaise C, Giroix MH, Cetik S, Malaisse WJ, Sener A (2013). Comparison of GLUT1, GLUT2, GLUT4 and SGLT1 mRNA expression in the salivary glands and six other organs of control, streptozotocin-induced and Goto-Kakizaki diabetic rats. Cell Physiol Biochem.

[CR19] Kinne R, Spokes KC, Silva P (2020). Sugar uptake, metabolism, and chloride secretion in the rectal gland of the spiny dogfish Squalus acanthias. Am J Physiol Regul Integr Comp Physiol.

[CR20] Kondo Y, Nakamoto T, Jaramillo Y, Choi S, Catalan MA, Melvin JE (2015). Functional differences in the acinar cells of the murine major salivary glands. J Dent Res.

[CR21] Lee JE, Nam JH, Kim SJ (2005). Muscarinic activation of Na^+^-dependent ion transporters and modulation by bicarbonate in rat submandibular gland acinus. Am J Physiol Gastrointest Liver Physiol.

[CR22] Lee MG, Ohana E, Park HW, Yang D, Muallem S (2012). Molecular mechanism of pancreatic and salivary gland fluid and HCO_3_^-^ secretion. Physiol Rev.

[CR23] Lin CC, Sun SS, Kao A, Lee CC (2002). Impaired salivary function in patients with noninsulin-dependent diabetes mellitus with xerostomia. J Diabetes Complications.

[CR24] Loo DD, Zeuthen T, Chandy G, Wright EM (1996). Cotransport of water by the Na^+^/glucose cotransporter. Proc Natl Acad Sci USA.

[CR25] Lytle C, Xu JC, Biemesderfer D, Forbush B (1995). Distribution and diversity of Na-K-Cl cotransport proteins: a study with monoclonal antibodies. Am J Physiol.

[CR26] Madunić IV, Breljak D, Karaica D, Koepsell H, Sabolić I (2017). Expression profiling and immunolocalization of Na^+^-D-glucose-cotransporter 1 in mice employing knockout mice as specificity control indicate novel locations and differences between mice and rats. Pflugers Arch.

[CR27] Malicka B, Kaczmarek U, Skośkiewicz-Malinowska K (2014) Prevalence of xerostomia and the salivary flow rate in diabetic patients. Adv Clin Exp Med 23:225–233. 10.17219/acem/37067 10.17219/acem/37067 24913113

[CR28] Melvin JE, Yule D, Shuttleworth T, Begenisich T (2005). Regulation of fluid and electrolyte secretion in salivary gland acinar cells. Annu Rev Physiol.

[CR29] Moore PA, Guggenheimer J, Etzel KR, Weyant RJ, Orchard T (2001). Type 1 diabetes mellitus, xerostomia, and salivary flow rates. Oral Surg Oral Med Oral Pathol Oral Radiol Endod.

[CR30] Nakamoto T, Srivastava A, Romanenko VG, Ovitt CE, Perez-Cornejo P, Arreola J, Begenisich T, Melvin JE (2007). Functional and molecular characterization of the fluid secretion mechanism in human parotid acinar cells. Am J Physiol Regul Integr Comp Physiol.

[CR31] Proctor GB (2016). The Physiology of Salivary Secretion Periodontol.

[CR32] Reynolds A, Parris A, Evans LA, Lindqvist S, Sharp P, Lewis M, Tighe R, Williams MR (2007). Dynamic and differential regulation of NKCC1 by calcium and cAMP in the native human colonic epithelium. J Physiol.

[CR33] Russell JM (2000). Sodium-potassium-chloride cotransport. Physiol Rev.

[CR34] Sabino-Silva R, Alves-Wagner AB, Burgi K, Okamoto MM, Alves AS, Lima GA (2010). SGLT1 protein expression in plasma membrane of acinar cells correlates with the sympathetic outflow to salivary glands in diabetic and hypertensive rats. Am J Physiol Endocrinol Metab.

[CR35] Silva P, Stoff J, Field M, Fine L, Forrest JN, Epstein FH (1977). Mechanism of active chloride secretion by shark rectal gland: role of Na-K-ATPase in chloride transport. Am J Physiol.

[CR36] Somasekharan S, Tanis J, Forbush B (2012). Loop diuretic and ion-binding residues revealed by scanning mutagenesis of transmembrane helix 3 (TM3) of Na-K-Cl cotransporter (NKCC1). J Biol Chem.

[CR37] Sugita M, Hirono C, Tanaka S, Nakahari T, Imai Y, Kanno Y (2000). Visualization of the secretory process involved in Ca^2+^-activated fluid secretion from rat submandibular glands using the fluorescent dye, calcein. Eur J Cell Biol.

[CR38] Sugita M, Hirono C, Shiba Y (2004). Gramicidin-perforated patch recording revealed the oscillatory nature of secretory Cl^-^ movements in salivary acinar cells. J Gen Physiol.

[CR39] Tarpey PS, Wood IS, Shirazi-Beechey SP, Beechey RB (1995). Amino acid sequence and the cellular location of the Na(+)-dependent D-glucose symporters (SGLT1) in the ovine enterocyte and the parotid acinar cell. Biochem J.

[CR40] Terachi M, Hirono C, Kitagawa M, Sugita M (2018). The biphasic effect of extracellular glucose concentration on carbachol-induced fluid secretion from mouse submandibular glands. Eur J Oral Sci.

[CR41] Zeuthen T, Meinild AK, Loo DD, Wright EM, Klaerke DA (2001). Isotonic transport by the Na^+^-glucose cotransporter SGLT1 from humans and rabbit. J Physiol.

